# Active clearance of chest tubes is associated with reduced postoperative complications and costs after cardiac surgery: a propensity matched analysis

**DOI:** 10.1186/s13019-019-0999-3

**Published:** 2019-11-08

**Authors:** Yvon Baribeau, Benjamin Westbrook, Yanick Baribeau, Simon Maltais, Edward M. Boyle, Louis P. Perrault

**Affiliations:** 1 Department of Cardiac Surgery, New England Heart and Vascular Institute, Catholic Medical Center, 100 McGregor St, Manchester, NH 03102 USA; 2 Department of Anesthesia and Critical Care, Beth Israel Deaconess Medical Center, Boston, MA USA; 3 Department of Cardiothoracic Surgery, Centre Hospitalier Universitaire de Montréal, Montreal, Canada; 4 Department of Thoracic Surgery, St. Charles Medical Center, Bend, OR USA; 5 Montreal Heart Institute, Montreal, Canada

**Keywords:** Chest tube, Cardiac surgery, Critical care, Postoperative atrial fibrillation, Pleural effusion, Costs

## Abstract

**Background:**

Chest tubes are routinely used to evacuate shed mediastinal blood in the critical care setting in the early hours after heart surgery. Inadequate evacuation of shed mediastinal blood due to chest tube clogging may result in retained blood around the heart and lungs after cardiac surgery. The objective of this study was to compare if active chest tube clearance reduces the incidence of retained blood complications and associated hospital resource utilization after cardiac surgery.

**Methods:**

Propensity matched analysis of 697 consecutive patients who underwent cardiac surgery at a single center. 302 patients served as a baseline control (Phase 0), 58 patients in a training and compliance verification period (Phase 1) and 337 were treated prospectively using active tube clearance (Phase 2). The need to drain retained blood, pleural effusions, postoperative atrial fibrillation, ICU resource utilization and hospital costs were assessed.

**Results:**

Propensity matched patients in Phase 2 had a reduced need for drainage procedures for pleural effusions (22% vs. 8.1%, *p* < 0.001) and reduced postoperative atrial fibrillation (37 to 25%, *P* = 0.011). This corresponded with fewer hours in the ICU (43.5 [24–79] vs 30 [24–49], p = < 0.001), reduced median postoperative length of stay (6 [4–8] vs 5 [4–6.25], *p* < 0.001) median costs reduced by $1831.45 (− 3580.52;82.38, *p* = 0.04) and the mean costs reduced by an average of $2696 (− 6027.59;880.93, 0.116).

**Conclusions:**

This evidence supports the concept that efforts to actively maintain chest tube patency in early recovery is useful in improving outcomes and reducing resource utilization and costs after cardiac surgery.

**Trial registration:**

Clinicaltrial.gov, NCT02145858, Registered: May 23, 2014.

## Background

In the early hours after heart surgery shed mediastinal blood accumulates until the postoperative bleeding stops. Shed mediastinal blood is evacuated by chest tubes positioned around the heart and lungs connected to external blood collection canisters set to suction. Chest tube clogging, however, can lead to un-evacuated shed mediastinal retained around the heart and lungs [1, 2]. This can contribute to the development of tamponade, hemothorax, or bloody pericardial and pleural effusions. Patients with retained blood have more complications including postoperative atrial fibrillation (POAF) and acute kidney injury (AKI) as well as utilization of more intensive care unit (ICU) resources and longer length of stay [1, 3–6]. In many cases, chest tubes are milked and stripped to attempt to clear the lumens of any clogging that can be visualized at the bedside [7]. However, these approaches have been shown to be ineffective and may be potentially harmful [8]. Active tube clearance (ATC) of drainage catheters was developed to allow a bedside mechanism for chest tube clearance of clot in the ICU during early recovery [9]. ATC has been shown to reduce interventions for retained blood as well as reduce the incidence of POAF during the index hospitalization after surgery [5, 10–12]. The purpose of this study was to determine if active tube clearance (ATC) might reduce the incidence of retained blood, as well as other associated hospital resources and costs after cardiac surgery.

## Materials and methods

This study included 639 consecutive patients undergoing adult cardiac surgery performed by two surgeons (YB and BW) at the New England Heart and Vascular Institute, Catholic Medical Center (CMC), Manchester, NH. The study protocol was registered (NCT02145858) and was approved by the CMC Institutional Review Board (CARD2015–6). Data was collected from our prospectively collected institutional Society of Thoracic Surgeons (STS) database. Additional analysis was supplemented with administrative data using ICD and CPT codes to obtain hospital costs. Patients were divided into those consecutive adult cardiac surgery patients who received conventional chest tubes only (Phase 0, *n* = 302), an ATC training and compliance verification segment (Phase 1, *n* = 58) and a consecutive cohort treated with ATC (Phase 2, *n* = 337). Aspirin was continued preoperatively, while thienopyridines (ADP inhibitors), and warfarin were generally held 5 to 7 days before the operation and GP IIb/IIIa were withheld within 1 day of surgery. In patients with preoperative ADP inhibitors needing urgent surgery, a thromboelastogram was performed and surgery allowed when the platelet inhibition level less than 50%. β–blockade was used in all suitable cases before and after the index procedure and amiodarone was not given prophylactically.

In all groups, all CABG patients were implanted with one 24 Fr chest tube in the retrosternal position, and one 24 Fr chest tube in the pleural space, if opened. All valve and other cases were implanted with 2 retrosternal 24 Fr chest tubes, and one in the pleural space, if opened. In Phase 0, when tube clots were identified, chest tube milking was carried out on an as needed basis at the nurses’ discretion. An ATC system (PleuraFlow Active Clearance Technology; ClearFlow, Inc., Anaheim, CA) was then introduced in Phase 1 and Phase 2. ATC is a chest tube clearance apparatus with a mechanism to actively keep the entire inner lumen of the chest tube clear of obstructing blood clot or fibrinous debris [9]. ATC was actuated every 15 min for the first 8 h, then every 30 min for the next 16 h, and then once an hour and as needed thereafter [5, 13]. No data was collected while the protocol was introduced and training completed (Phase 1). Other than the use of ATC in Phase 2, there were no other differences in the size, placement location or management of chest tubes during this study. Mediastinal chest tubes were removed 24 h postoperatively or when drainage volume was less than 50 mL during the previous 8 h. Pleural chest tubes were removed 48 h postoperatively or when drainage volume was less than 200 mL during the past 24 h.

Phase 0 patients were then compared with Phase 2 for the primary endpoint of retained blood interventions after the index procedure. Retained blood is a previously described composite endpoint that encompasses any postoperative re-intervention to treat one or more of the following in the first 30 days after an initial index procedure: Re-exploration for bleeding with washout of retained blood, pleural interventions-percutaneous (thoracentesis or supplemental chest tube placed after surgery), pleural interventions-surgical (thoracotomy or thoracoscopy for hemothorax after surgery), or pericardial interventions (pericardial window or pericardiocentesis) [1, 4, 5, 11]. All patients were monitored continuous telemetry from index surgery for 3 days in CABG only patients, and through hospital discharge for all other patients. Consistent with the STS definition, POAF was defined as an episode of atrial fibrillation/flutter lasting longer than one hour and/or requiring treatment at any time between post index surgery through hospital discharge. Patients who had a documented history of atrial fibrillation/flutter prior to surgery were excluded from the POAF sub analysis. Acute Kidney Injury (AKI) was defined by KDIGO stage [14]. Chest tube output was measured on an hourly basis until chest tube removal. Hospital costs were provided by the hospital’s finance department. Cost were defined as total cost data for Phase 0 and Phase 2 subjects from the time of admission to the time of discharge.

### Statistical analysis

Table 1 describes the summary statistics, standardized difference calculation, and the statistical tests used for each variable*.* The non-symmetrically distributed continuous variables tended to have long right tails, and so the analysis calculated standardized differences after log-transforming these variables. The subscripts 0 and 2 refer to Phase 0 and Phase 2 and the symbols $\overline{X},s,m,{q}_1,{q}_3,c,f,$ and $\hat{p}$ refer to the following sample statistics: mean, standard deviation, median, first quartile, third quartile, total count, frequency, and proportion. The analysis matched the patients from Phase 0 and Phase 2 in a one-to-one fashion using the *pairmatch* function from the R package *optmatch*. This matching combined propensity score matching based on the variables given in Table 2 with forced matching on ADP inhibitors, glycoprotein IIb/IIIa inhibitors, and procedure type. Hospital cost data are reported as both median and mean values. Confidence intervals for the cost differences between Phase 2 and Phase 0 were obtained via bootstrapping for the mean and via quantile regression for the median. A two-sided *p*-value ≤0.05 was considered statistically significant.

**Table 1:** Summary of statistical methods based on the types of variables

Type of variable *X*	Summary statistics	Standardized difference	Statistical test for unmatched data	Statistical test for matched data
Symmetric continuous variable	$\overline{X}\pm s$	$\frac{{\overline{X}}_2-{\overline{X}}_0}{\sqrt{\left({s}_2^2+{s}_0^2\right)/2}}$	Independent samples t-test	Paired samples t-test
Non-symmetric continuous variable	*m* [*q*_1_ − *q*_3_]	The standardized difference defined above for ln*X*	Wilcoxon’s rank sum test	Wilcoxon’s signed rank test
Count variable	*c*	Standardized difference for symmetric continuous variable	Independent samples t-test	Paired samples t-test
Binary variable	$f\ \left(\hat{p}\%\right)$	$\frac{\hat{p_2}-\hat{p_0}}{\sqrt{\left(\hat{p_2}\left(1-\hat{p_2}\right)+\hat{p_0}\left(1-\hat{p_0}\right)\right)/2}}$	Fisher’s exact test	McNemar’s test

**Table 2:** Demographic and Pre/Intra-operative Characteristics (Unmatched)

Variable (mean n, %)	All (*n* = 639)	P0 (*n* = 302)	P2(*n* = 337)	*p* value
Age (years)	66 ± 11.6	67 ± 10.2	65.1 ± 12.6	0.036
Gender (male)	441 (69%)	205 (68%)	236 (70%)	0.61
Weight (kg)	89 ± 20	88 ± 20	90 ± 21	0.13
Diabetes	274 (43%)	118 (39%)	156 (46%)	0.078
Hypertension	515 (81%)	243 (80%)	272 (81%)	1
Prior PCI	155 (24%)	70 (23%)	85 (25%)	0.58
NYHA I	329 (51%)	162 (54%)	167 (50%)	0.3
NYHA II, III or IV	310 (49%)	140 (46%)	170 (50%)	0.3
Preop atrial arrhythmia	131 (21%)	59 (20%)	72 (21%)	0.62
Preop-IABP	50 (7.8%)	25 (8.3%)	25 (7.4%)	0.77
ADP inhibitors	55 (8.6%)	37 (12%)	18 (5.3%)	0.0027
Preop Aspirin	621 (97%)	300 (99%)	321 (95%)	0.0015
Preop Warfarin	6 (0.94%)	3 (0.99%)	3 (0.89%)	1
GIIb/IIIa	7 (1.1%)	7 (2.3%)	0 (0%)	0.0051
First Time Surgery	601 (94%)	284 (94%)	317 (94%)	1
Reoperation	38 (5.9%)	18 (6%)	20 (5.9%)	1
Operative Status (Elective)	269 (42%)	130 (43%)	139 (41%)	0.69
Opertive Status (Non Elective)	370 (58%)	172 (57%)	198 (59%)	0.69
CABG	359 (56%)	167 (55%)	192 (57%)	0.69
Valve	79 (12%)	49 (16%)	30 (8.9%)	0.0056
CABG + Valve	78 (12%)	38 (13%)	40 (12%)	0.81
Other	123 (19%)	48 (16%)	75 (22%)	0.045
CPB (yes)	295 (46%)	140 (46%)	155 (46%)	0.94

Abbreviations: Coronary Artery Bypass Surgery (CABG), Cardiopulmonary Bypass (CPB), Glycoprotein GIIb/IIIa inhibitor (GIIb/IIIa), Intra Aortic Balloon Pump (IABP), New York Heart Association (NYHA), Percutaneous Cardiac Interventions (PCI)

## Results

Unmatched patient characteristics and outcomes are presented in Tables 2, 3 and 4. After propensity matching, 260 of 302 patients in Phase 0 were selected for comparison with 260 matched patients in Phase 2. The propensity matching balanced the Phase 0 and Phase 2 patients with respect to the demographic and pre/intra-operative characteristics (Table 5). In Phase 2 more than 1 ATC were used in select cases resulting in an average of 1.2 ATC inserted per patient in the retrosternal position over an open pericardium. The overall incidence of retained blood in matched Phase 0 patients through the first 30 days at baseline (Phase 0) was 23% (60/260), with a 59% reduction to 9.6% (25/260) in the matched Phase 2 patients (*P* < 0.001). (Table 6) The number needed to treat for this RBS reduction was 7.4 [95% CI 5–14.6, *p* = 0.001} The rate of percutaneous pleural interventions was similarly reduced in the analysis from 22% in Phase 0 to 8.1% (P < 0.001) in Phase 2. Patients in Phase 2 had a reduced number of pleural interventions. (139 vs 53, *p* = 0.002). The other less common components of the retained blood composite such as re-exploration for bleeding, pericardial drainage procedures, surgical pleural drainage procedures were not significantly different between phases. Phase 2 patients had a 32.4% reduced incidence of POAF (37% in Phase 0 to 25% in Phase 2; *p* = 0.011) (Table 5). The NNT for the reduction in POAF was 8.7 [CI 4.9–43.6, *p* = −.014] There was a reduction in KDIGO stage 1 AKI from 30 to 17% (*P* = 0.0014) and total chest tube output was significantly lower in Phase 2. (1152 ml vs 1037.50 mL, *p* = 0.02) There were no statistically significant reductions in hospital mortality, cardiac arrest, or permanent stroke. (Table 6) Additionally, there was a marked reduction total ICU hours (43 [24–79] vs 29.75 [24–49], *p* < 0.001); ICU stay > 3 days 27% vs 12%, < 0.001), time on the ventilator in hours (9.1 [5–20] vs 7.3 [4.9–17.2], p 0.004). (Table 7) Postoperative length of stay was reduced by 1 day from 6 [4–8] to 5 [4–6.25], (*P* < 0.001). The matched median costs (Table 8) were reduced by -$1831.45 ([−$3580.52; $82.38], *p* = 0.04) the mean costs (Table 9) reduced by an average of -$2696 ([−$6027.59; $880.93], *p* = 0.116).

**Table 3:** Postoperative Outcomes (Unmatched)

Variable (mean or mean, n, %)	P0 (*n* = 302)	P2 (*n* = 337)	*p* value
RB (composite)	72 (24%)	31 (9.2%)	< 0.001
Re-exploration	6 (2%)	3 (0.89%)	0.32
Pleural Intervention-Percutaneous	69 (23%)	25 (7.4%)	< 0.001
Pleural Intervention- Surgical	2 (0.66%)	1 (0.3%)	0.6
Pericardial Intervention	4 (1.3%)	4 (1.2%)	1
# Pleural Effusion drained	168	59	0.001
POAF	93 (37%) (*n* = 251)	67 (25%) (*n* = 270)	0.0032
Cardiac arrest	8 (2.6%)	8 (2.4%)	1
Postoperative blood products used	72 (24%)	61 (18%)	0.079
CTO (mL) first 24 h	740 [561.3–975]	680 [532–910]	0.016
Total CTO (mL)	1136 [834.3–1645]	1040 [760–1430]	0.041
Total CT (days)	3 [3–4]	2 [2–2]	< 0.001
Infection (any)	39 (13%)	14 (4.2%)	< 0.001
Surgical site deep	0 (0%)	0 (0%)	1
Surgical site superficial	0 (0%)	0 (0%)	1
Surgical site mediastinitis	0 (0%)	0 (0%)	1
Pneumonia	20 (6.6%)	6 (1.8%)	0.0022
Septicemia	4 (1.3%)	0 (0%)	0.049
UTI	11 (3.6%)	4 (1.2%)	0.064
C. Diff	3 (0.99%)	2 (0.59%)	0.67
Highest postop- creatinine	1.1 [0.905–1.4]	1 [0.83–1.33]	< 0.001
AKI Stage 1	85 (28%) (*n* = 299)	67 (20%) (*n* = 332)	0.02
AKI Stage 2	8 (2.7%)	8 (2.4%)	1
AKI Stage 3	4 (1.3%)	3 (0.9%)	0.71
Postop-renal dialysis	7 (2.3%)	2 (0.59%)	0.092
Stroke	2 (0.66%)	4 (1.2%)	0.69
Mortality	7 (2.3%)	9 (2.7%)	0.81

Abbreviations: Retained Blood (RB), Postoperative atrial fibrillation (POAF); Chest tube output (CTO); Chest tube (CT), Urinary Tract Infection (UTI), *Clostridium difficile* (C.Diff), Acute Kidney Injury (AKI)

**Table 4:** ICU and Hospital Length of Stay (unmatched)

Variable (median or mean %)	P0 (*n* = 302)	P2 (*n* = 337)	*p* value
ICU time (Hrs.)	44.5 [24–83]	30 [24–49]	< 0.001
ICU stay > 3 days	82 (27%)	43 (13%)	< 0.001
Total time on ventilator (Hrs.)	8.7 [5–19.7]	8.2 [5.0–17.5]	0.27
Ventilation > 24 h.	49 (16%)	39 (12%)	0.11
Hospital LOS (d)	8 [5–11]	7 [5–10]	0.017
Post-op LOS (d)	6 [4–8]	5 [4–7]	< 0.001

Abbreviations: Intensive Care Unit (ICU), Hours (Hrs), Length of Stay (LOS), Postoperative (Post-op); days (d)

**Table 5:** Demographic, Preoperative and Intraoperative Characteristics (Matched)

Variable (mean n, %)	Phase 0 (*n* = 260)	Phase 2 (*n* = 260)	*p* value
Age (years)	66.7 ± 10.1	66 ± 11.6	.4
Gender (male)	177 (68%)	177 (68%)	1
Weight (kg)	88 ± 21	90 ± 21	.33
Diabetes	106 (41%)	107 (41%)	1
Hypertension	209 (80%)	213 (82%)	.73
Prior PCI	61 (23%)	66 (25%)	.67
NYHA Class I	139 (53%)	133 (51%)	.63
NYHA Class II, III or IV	121 (47%)	127 (49%)	.63
Preoperative atrial arrhythmia	51 (20%)	49 (19%)	.91
Preoperative IABP	19 (7.3%)	22 (8.5%)	.74
ADP inhibitors	18 (6.9%)	18 (6.9%)	1
Preoperative Aspirin	258 (99%)	257 (99%)	1
Preoperative Warfarin	3 (1.2%)	3 (1.2%)	1
GIIa/IIIa	0 (0%)	0 (0%)	1
First Time Surgery	244 (94%)	246 (95%)	.86
Reoperation	16 (6.2%)	14 (5.4%)	.86
Operative Status (Elective)	112 (43%)	117 (45%)	.71
Operative Status (Non-elective)	148 (57%)	143 (55%)	.71
CABG	147 (57%)	154 (59%)	.54
Valve	30 (12%)	30 (12%)	1
CABG + valve	35 (13%)	32 (12%)	.78
Other procedure	48 (18%)	44 (17%)	.7
CPB (yes)	114 (44%)	119 (46%)	.69

Abbreviations: Coronary Artery Bypass Surgery (CABG), Cardiopulmonary Bypass (CPB), Glycoprotein GIIb/IIIa inhibitor (GIIb/IIIa), Intra Aortic Balloon Pump (IABP), New York Heart Association (NYHA), Percutaneous Cardiac Interventions (PCI)

**Table 6:** Postoperative Outcomes (Matched)

Variable (mean or mean, n, %)	Phase 0 (*n* = 260)	Phase 2(*n* = 260)	*p* value
Retained Blood (composite)	60 (23%)	25 (9.6%)	<.001
Re-exploration	6 (2.3%)	2 (0.77%)	.29
Pleural Intervention-Percutaneous	57 (22%)	21 (8.1%)	<.001
Pleural Intervention- Surgical	2 (0.77%)	0 (0%)	0.25
Pericardial Intervention	4 (1.5%)	4 (1.5%)	1
# Pleural Effusions drained	139	53	.002
POAF	79 (37%) (*n* = 214)	55 (25%) (*n* = 216)	.011
Cardiac arrest	8 (3.1%)	5 (1.9%)	.58
Postoperative blood products used	60 (23%)	48 (18%)	.2
CT output (mL) first 24 h	753 [584–975]	678 [539–950]	.038
Total CT output (mL)	1152 [854–1656]	1038 [760–1431]	.020
Total CT (days)	3 [3–4]	2 [2–2]	<.001
Infection (any)	37 (14%)	10 (3.8%)	<.001
Surgical site deep	0 (0%)	0 (0%)	1
Surgical site superficial	0 (0%)	0 (0%)	1
Surgical site mediastinitis	0 (0%)	0 (0%)	1
Pneumonia	20 (7.7%)	4 (1.5%)	.0022
Septicemia	4 (1.5%)	0 (0%)	.062
UTI	10 (3.8%)	4 (1.5%)	.18
C. Diff	3 (1.2%)	1 (0.38%)	.62
Highest postoperative creatinine	1.1 [1–1.4]	1.0 [0.8–1.2]	<.001
AKI Stage 1	78 (30%) (*n* = 257)	44 (17%) (*n* = 256)	.0014
AKI Stage 2	7 (2.7%) (n = 257)	4 (1.6%) (n = 256)	.55
AKI Stage 3	3 (1.2%) (n = 257)	1 (0.39% (n = 256)	.62
Postoperative renal dialysis	7 (2.7%)	0 (0%)	.0078
Stroke	2 (0.77%)	2 (0.77%)	1
Mortality	7 (2.7%)	7 (2.7%)	1

Abbreviations: Postoperative atrial fibrillation (POAF); Chest tube (CT), Urinary Tract Infection (UTI), *Clostridium difficile* (C.Diff), Acute Kidney Injury (AKI),

**Table 7:** ICU and Hospital Length of Stay (Matched)

Variable (median or mean n, %)	Phase 0 (*n* = 260)	Phase 2 (*n* = 260)	*p* value
Total ICU time (Hrs.)	43.5 [24–79.25]	29.75 [24–49]	<.001
ICU stay > 3 days	70 (27%)	31 (12%)	<.001
Total time on ventilator (Hrs.)	9.1 [5–20]	7.3 [4.9–17.2]	.004
Ventilation > 24 h	44 (17%)	27 (10%)	.037
Total hospital length of stay (d)	7.5 [5–11]	7 [5–10]	.045
Total post-op length of stay (d)	6 [4–8]	5 [4–6.25]	<.001

Abbreviations: Intensive Care Unit (ICU), Hours (Hrs), Length of Stay (LOS), Postoperative (Post-op); days (d)

**Table 8:** Median Index Hospitalization Costs by Department, using Quantile Regression (Matched)

Department	P0	Costs (95% CI)		
		P2	Difference (P2-P0)	*p* value
Cardiology	$9.74 (7.29, 12.19)	$13.20 (10.57, 15.83)	$3.46 (−0.13, 7.05)	0.0591
Laboratory	$968.86 (881.95, 1055.77)	$734.63 (665.03, 804.23)	-$234.23 (− 345.58, − 122.88)	< 0.0001
Other	$1112.88 (1044.25, 1181.51)	$1764.89 (1733.14, 1796.64)	$652.01 (576.39, 727.63)	< 0.0001
Radiology	$378.97 (335.58, 422.36)	$264.36 (239.27, 289.45)	-$114.61 (−164.73, −64.49)	< 0.0001
Step Down/Ward	$2591.52 (2426.63, 2756.41)	$2621.32 (2455.39, 2787.25)	$29.80 (− 204.13, 263.73)	0.8025
Respiratory	$420.71 (341.32, 500.10)	$250.22 (212.49, 287.95)	-$170.49 (− 258.39, −82.59)	0.0002
ICU	$2710.08 (2369.00, 3051.16)	$1782.60 (1420.70, 2144.50)	-$927.48 (− 1424.78, −430.18)	0.0003
Operating Room - Index	$12,514.12 (11,829.12, 13,199.12)	$12,395.89 (11,907.61, 12,884.17)	-$118.23 (− 959.44, 722.98)	0.7826
Blood Bank	$120.14 (71.77, 168.51)	$73.10 (51.45, 94.75)	-$47.04 (−100.04, 5.96)	0.0818
OR/Anesthesia	$525.61 (516.73, 534.49)	$511.53 (503.17, 519.89)	-$14.08 (−26.27, −1.89)	0.0237
Pharmacy	$2181.44 (2010.88, 2352.00)	$1430.57 (1325.98, 1535.16)	-$750.87 (−950.94, − 550.80)	< 0.0001
Total	$25,072.80 (23,662.97, 26,482.63)	$23,241.35 (22,206.16, 24,276.54)	-$1831.45 (−3580.52, −82.38)	0.0402

**Table 9:** Mean Index Hospitalization Costs ($) by Department, with 95% CI from bootstrapping (Matched)

Department	Costs (95% CI)			*p* value
	P0	P2	Difference (P2-P0)	
Cardiology	$323.00 (177.44, 498.57)	$457.36 (202.86, 780.58)	$134.36 (− 194.43, 491.74)	0.466
Laboratory	$1111.94 (1021.96, 1203.00)	$900.76 (831.97, 975.97)	-$211.18 (− 316.80, −99.11)	< 0.0001
Other	$1508.14 (1377.42, 1658.83)	$1875.89 (1767.38, 2005.89)	$367.75 (169.65, 550.74)	< 0.0001
Radiology	$1018.82 (844.36, 1206.31)	$577.06 (473.02, 702.34)	-$441.75 (− 654.92, −234.25)	< 0.0001
Step Down/Ward	$2955.18 (2724.31, 3,223.20)	$3012.16 (2685.25, 3,387.11)	$56.98 (− 346.77, 484.92)	0.760
Respiratory	$856.63 (678.98, 1037.94)	$569.12 (440.17, 730.86)	-$287.51 (−511.17, −46.08)	0.020
ICU	$4887.07 (4020.89, 5802.31)	$3919.07 (3110.13, 4956.25)	-$968.00 (− 2199.04, 365.86)	0.156
Operating Room	$14,024.07 (13,528.39, 14,538.16)	$13,631.57 (13,086.79, 14,125.95)	-$392.50 (− 1028.30, 309.46)	0.244
Blood Bank	$811.35 (616.39, 1022.17)	$571.44 (393.92, 776.12)	-$239.91 (− 510.83, 31.15)	0.088
OR/Anesthesia	$548.80 (536.12, 562.48)	$531.82 (521.51, 543.23)	-$16.97 (−33.86, 0.99)	0.074
Pharmacy	$3175.06 (2723.25, 3713.31)	$2259.84 (1858.48, 28,18.09)	-$915.22 (− 1595.18, − 196.74)	0.014
Total	$31,549.19 (29,149.07, 34,144.06)	$28,852.59 (26,677.52, 31,341.75)	-$2696.59 (−6027.70, 880.93)	0.116

## Discussion

Chest tubes are used routinely in the intensive care setting to clear blood and air from the mediastinal and pleural spaces after cardiac and thoracic surgery and trauma. Chest tube clogging can develop when shed blood comes in contact with the inside of drainage catheters, occurring in up to 36% of prospectively evaluated heart surgery patients [2, 15]. Chest tube stripping, milking and open suction have been utilized in an attempt to prevent chest tube clogging, but its safety and efficacy are not shown when reviewed in meta-analysis of randomized trials [8]. In particular, a major issue for chest tube stripping and milking is the potential for the high negative pressures generated to injure the heart or great vessels, further exacerbating bleeding. Thus alternatives are needed to allow protocols to be implemented to prevent chest tube clogging. ATC was developed as a low risk alternative to these approaches [9]. In preclinical studies ATC was more effective in evacuating blood, even when smaller diameter tubes were compared to larger diameter tubes [16, 17]. Clinically ATC is readily integrated into the operating room and ICU without disruption of the usual workflow [13].

To quantify the clinical sequelae of chest tube clogging, a composite clinical endpoint of retained blood interventions was proposed in prior publications [1, 3–5]. Retained blood as an endpoint is defined as any postoperative intervention to remove blood, blood clot or bloody fluid after cardiac surgery [4]. This includes taking a patient back to wash out mediastinal clot or hemothorax, inserting an additional chest tube in the ICU, thoracentesis or pericardial drainage for bloody effusions. Sirch, et al., previously demonstrated a 43% relative risk reduction in retained blood in an all comers population of cardiac surgery patients treated with ATC, and Maltais, et al., reported a 59% reduction in retained blood in a mechanical assist cohort of patients [5, 11]. Likewise in this study, we noted a 59% relative risk reduction in retained blood in matched patients.

The primary driver of the reduction in the retained blood composite endpoint were interventions to drain pleural effusion by thoracentesis. In this series over the course of 30 days, 22% of Phase 0 patients required at least one pleural intervention which was reduced to 8.1% in patients in Phase 2. The majority of early pleural effusions are known to be bloody and inflammatory after cardiac surgery [18, 19]. By minimizing retained blood in the pericardium and pleural spaces there could be less of an inflammatory process driving fluid exudation from the inflamed mesothelium during recovery [4]. Although there was a trend toward reduced take back for re-exploration for bleeding for washout of mediastinal clot the sample sizes was too small for statistical comparison [20].

In this study, as also seen by Sirch, et al., the volume of bleeding (measured as chest tube output) was significantly less in Phase 2 patients at 24 h and in total [5]. While this may seem counter intuitive, it suggest there may be an advantage of more rapidly clearing shed mediastinal blood to prevent on going microvascular bleeding from the cut surfaces in the early hours after surgery. Tissue plasminogen activator (t-PA) is known to significantly accumulate in shed mediastinal blood, which can promote on going microvascular bleeding within the postsurgical space if not promptly evacuated by chest tubes [21]. Therefore perhaps having shed mediastinal blood more effectively evacuated could leave less t-PA remaining in contact with these tissues, facilitating a more rapid achievement of microvascular hemostasis in this time period.

Retained blood is recognized as an important trigger for POAF in susceptible individuals after heart surgery [22–24]. Blood not evacuated from the pericardial space coagulates, generating thrombin that recruits neutrophils which promote a localized inflammatory response on the surface of the atrium that is highly pro-oxidant and inflammatory [6]. In patients who have susceptible atrial substrate this oxidative response can trigger POAF [23]. In the present study, there was a 32.4% reduction in POAF. This is consistent with the studies by Sirch, where patients treated with ATC had a 30% reduction in POAF, and St. Onge, where there was a 34% reduction in POAF [5, 12]. This could have a significant impact on hospital resource utilization, as POAF is known to significantly increase hospital costs [25].

AKI is a well-recognized complication of cardiac surgery in the postoperative period [14]. In this study, patients with ATC had lower postoperative creatinine levels as well as a significant reduction in the need for postoperative dialysis. The calculated KDIGO stage I AKI rate was 30% during Phase 0, controls, dropping to 17% in Phase 2 (*P* = 0.0014). There was a trend towards a reduction in KDIGO stage II and III AKI, but the sample sizes were too small for this analysis. This study was not designed to specifically study these endpoints or the mechanism by which this may occur. However, several mechanisms can be considered such as a reduction in hypotension from tamponade or hypoxia from effusions might put less stress on the kidneys during recovery. Additionally, perhaps if there is less retained blood there is less reabsorption of oxidized products of which are known to contribute to AKI [26]. AKI imposes a sizable financial burden for hospitals and thus reducing this outcome could contribute to a reduction of overall hospital costs [27]. Further studies are indicated to more specifically evaluate this endpoint and the possible mechanisms of how ATC might be an additional adjunct to help minimize AKI after heart surgery.

Patients with ATC had a reduction in hospital resource utilization including a reduction in ICU hours, ICU stays of over 3 days, and reduced incidence of prolonged intubation (defined as > 24 h). There was a statistically significant reduction in infections, primarily driven by a reduction in pneumonia and sepsis. Patients in Phase 2 had a 1 day reduced total and hospital postoperative length of stay, again, which could have significant hospital resource use implications (measured in costs, not charges) [5, 12]. This suggests that ATC is economically justifiable for hospitals, even after including the costs of acquiring the technology. In the United States, ATC costs approximately $395 per unit, and in this study it was implemented as a preventative measure in all patients. Our program performs 700 cardiac cases on a yearly basis. Assuming that 700 cases a year utilizing 1.2 ATC per case, the yearly costs to implement ATC is $331,800. A median net savings of $1831 per patient over 700 patients would result in a net recuperation of $1,281,700 per year for the hospital. A mean net savings of $2696 per patient over 700 patients would result in a net recuperation of $1,887,200 per year for the hospital. Both of these sums provide a considerable margin for cost effectiveness from a hospital purchasing perspective. This important administrative consideration is crucial in the current healthcare environment which endeavors to increase healthcare value by reducing complications and costs [28, 29].

There are several limitations to this study. First, data were generated from a nonrandomized, prospectively collected observational cardiac surgical database supplemented by retrospective chart reviews. These cases, however, represented a 100% census of all cardiac surgical procedures occurring during the duration of this study, which could have limited the potential for selection bias. Second, the endpoint of retained blood relies on the analysis of patients who had interventions performed, rather than by direct imaging for retained blood. This has the disadvantage of being more dependent on the subjective decision of the operating surgeon to intervene to evacuate retained blood. On the other hand, this strictly represents the experience of only two operating surgeons who have internally consistent practice patterns. Imaging may be more likely to include retained blood of uncertain clinical significance, while relying on the definition that requires specific invasive intervention for retained blood may represent a more clinically meaningful endpoint with quality improvement ramifications. Finally, although we implemented an ATC protocol for all patients undergoing cardiac surgery in our program, it is important to note that sub populations of patients such as those having CABG only, on pump vs off pump CABG, valve surgery, reoperations and more complex combined procedures have different risks and patterns of postoperative bleeding and thus may have differing clinical responses to such a clinical protocol. Further studies are needed that are statistically powered to examine these different cardiac surgery populations so that the outcomes with this approach can be better defined and protocols more specifically developed to best serve these patients.

## Conclusion

The use of ATC is associated with a reduction in retained blood, POAF, AKI, blood loss, ICU and hospital resource utilization and median costs. This supports the concept that efforts to actively maintain chest tube patency in the ICU after heart surgery improve outcomes and are economically justified to help reduce hospital resource utilization after cardiac surgery.

## Data Availability

Data are available upon request.

## Abbreviations

$: US Dollar
ADP: Adenosine diphosphate receptor inhibitors
AKI: Acute kidney injury
ATC: Active tube clearance
C Diff: *Clostridium difficile* Infection
CABG: Coronary Artery Bypass Surgery
CPT: Current procedural terminology code
CT: Chest tube
CTO: Chest tube output
d: day
GP: Glycoprotein
Hrs: Hours
IABP: Intra aortic balloon pump
ICD: International classification of diseases code
ICU: Intensive Care Unit
KDIGO: Kidney Disease Improving Global Outcomes
LOS: Length of Stay
NYHA: New York Heart Association
PCI: Percutaneous Intervention
POAF: Post-operative atrial fibrillation
POD: Postoperative day
t-PA: Tissue plasminogen activator
UTI: Urinary tract infection
